# Supplementary material for: Assessing Discharge Readiness After Propofol-Mediated Deep Sedation in Pediatric Dental Procedures: Revisiting Discharge Practices with the Modified Aldrete Recovery Score
Source: Children (Basel). 2025 Aug 29;12(9):1155. doi: 10.3390/children12091155 (PMC12469054; doi:10.3390/children12091155)
Supplement: Supplementary file 1 [file children-12-01155-s001.zip › children-3823323-supplementary.pdf]

Supplementary Material

Table S1. American Society of Anesthesiologists (ASA) Physical Status Classification System.

Class	Definition
ASA I	A healthy patient.
ASA II	A patient with mild systemic disease (no substantive functional limitations).
ASA III	A patient with severe systemic disease (substantive functional limitations).
ASA IV	A patient with severe systemic disease that is a constant threat to life.
ASA V	A moribund patient who is not expected to survive without the operation.
ASA VI	A declared brain-dead patient whose organs are being removed for donor purposes.

*Add the suffix 'E' for emergency procedures (e.g., ASA IIIE).
(www.ncbi.nlm.nih.gov/books/NBK441940/)
